# Overexpression of Nitrate Transporter 1/Peptide Gene *OsNPF7.6* Increases Rice Yield and Nitrogen Use Efficiency

**DOI:** 10.3390/life12121981

**Published:** 2022-11-26

**Authors:** Min Zhang, Liuru Lai, Xintong Liu, Jiajia Liu, Ruifang Liu, Yamei Wang, Jindong Liu, Jingguang Chen

**Affiliations:** 1 College of Pharmacy, Henan University of Chinese Medicine, Zhengzhou 450046, China; 2 School of Agriculture, Shenzhen Campus, Sun Yat-sen University, Shenzhen 518107, China; 3 Shandong Jinchunyu Seed Technology Co., Ltd., Jining 272200, China; 4 The High School Affiliated to Renmin University of China, Shenzhen 518119, China; 5 Institute of Crop Sciences, National Wheat Improvement Center, Chinese Academy of Agricultural Sciences (CAAS), 12 Zhongguancun South Street, Beijing 100081, China; 6 Shenzhen Branch, Guangdong Laboratory for Lingnan Modern Agriculture, Genome Analysis Laboratory of the Ministry of Agriculture, Agricultural Genomics Institute, Chinese Academy of Agricultural Sciences, Shenzhen 518120, China

**Keywords:** ^15^NO_3_^−^ influx, agronomic nitrogen use efficiency, functional analysis, *OsNPF7.6*, *Oryza sativa*

## Abstract

Overuse of nitrogen fertilizer in fields has raised production costs, and caused environmental problems. Improving nitrogen use efficiency (NUE) of rice is essential for sustainable agriculture. Here we report the cloning, characterization and roles for rice of *OsNPF7.6*, a member of the nitrate transporter 1/peptide transporter family (NPF). The OsNPF7.6 protein is located in the plasma membrane, expressed in each tissue at all stages and is significantly regulated by nitrate in rice. Our study shows that the overexpression of *OsNPF7.6* can increase the nitrate uptake rate of rice. Additionally, field experiments showed that *OsNPF7.6* overexpression increased the total tiller number per plant and the grain weight per panicle, thereby improving grain yield and agronomic NUE in rice. Thus, *OsNPF7.6* can be applied to be a novel target gene for breeding rice varieties with high NUE, and provide a reference for breeding higher yielding rice.

## 1. Introduction

Nitrogen is one of the most crucial macronutrients for crops and a sufficient nitrogen supply can ensure plant development and high yield [1,2,3,4]. Nitrogen fertilization is the key factor to improve crop yield and reduce hunger worldwide [5]. In an effort to boost productivity, farmers typically apply an excessive amount of nitrogen fertilizer, but less than 50% of it is actually absorbed by the plants, and the remnant ends up polluting the environment [6]. Improving rice (*Oryza sativa* L.) nitrogen use efficiency (NUE) offers an effective and practical way to increase grain yield and solve the environmental issues caused by excessive nitrogen application.

NO_3_^−^ and NH_4_^+^ are two main sources of inorganic nitrogen during crop growth. Rice makes up about 25% of all calories consumed globally [7,8,9]. Conventionally grown rice is submerged in water, which inhibits nitrification and makes NH_4_^+^ the most significant inorganic nitrogen in rhizosphere soil. Based on this, rice is regarded as an ammonium-preferring plant [10]. On the other hand, rice can release some oxygen produced by photosynthesis to the rhizosphere through roots due to its mature aerial tissue [11]. Because oxygen promotes the growth and propagation of nitrifying bacteria, partial NH_4_^+^ in the rhizosphere can be converted to NO_3_^−^ [12]. Consequently, during rice growth, its root system is under the mixed nutrition of NO_3_^−^ and NH_4_^+^ [13].

Previous studies have established that the prominent families of transporters involved in nitrate-nitrogen uptake and transport in plants include nitrate transporter1/peptide transporter family (NPF) and nitrate transporter 2 (NRT2). The *NRT2* family encodes high-affinity NO_3_^−^ transport proteins, which have essential functions in response to low-nitrogen (NO_3_^−^) environments [14]. Among the members of NRT2s, OsNRT2.1, OsNRT2.2 and OsNRT2.3a require the assistance of OsNAR2.1 to complete the transport of nitrate [15]. The interaction of OsNAR2.1 with OsNIT1 and OsNIT2, affects the uptake of nitrate and ammonium by rice roots [16]. *OsNRT2.3* yields two transcripts including *OsNRT2.3a* and *OsNRT2.3b* [17]. *OsNRT2.3a* plays a role in the long-distance transport of nitrate from roots to aerial parts [18], while *OsNRT2.3b* can elevate the pH buffering ability of rice [19]. Compared with all other *NRT2* genes, OsNRT2.4 is a dual-affinity nitrate transporter, which is necessary for nitrate-regulation of root and shoot growth of rice [20].

The *NPF* family includes two genes, *NRT1* (*nitrate transporter1*) and *PTR* (*peptide transporter*). The former is generally believed to encode the low-affinity NO_3_^−^ transporter, and the latter encodes the oligopeptide transporter [21]. Due to the high sequence similarity of the two members, in the same branch of evolutionary relationship, the *NRT1/PTR* gene is uniformly named *NPF* (*NRT1/PTR family*) [22]. There are many members of the *NPF* family, and greater focus and thorough investigation have been given to the NPF genes’ roles. Numerous studies have demonstrated that NPF genes are widely implicated in the uptake and utilization of nitrogen in plants and have essential roles and applications in improving nitrogen utilization and enhancing yield-related traits [23,24,25,26,27,28]. With the use of the homologous sequence of Arabidopsis *AtNRT1.1, OsNRT1.1* has been cloned in rice [29]. Two varying splicing forms including *OsNRT1.1a* and *OsNRT1.1b* are shown in *OsNRT1.1*. *OsNRT1.1a* only acts under high-nitrogen situations, while *OsNRT1.1b* is found to facilitate nitrogen uptake under low- nitrogen situations [30]. Moreover, overexpression of *OsNRT1.1A* could increase NUE [25]. A single-base variation of *NRT1.1B* explains the difference in NUE between *indica* and *japonica* subspecies [26]. The NADH/NADPH-dependent nitrate reductase gene OsNR2 can interact with *OsNRT1.1b*, and promote the uptake of nitrate in *indica* rice [31]. OsNPF6.1 serves as a dual-affinity nitrate transporter, the transcription factor OsNAC42 could activate OsNPF6.1, which can thus elevate rice NUE [32].

Here, we report that the nitrate transporter OsNPF7.6 is induced by nitrate, and overexpression of *OsNPF7.6* increases nitrate uptake rate, yield, as well as NUE in rice.

## 2. Materials and Methods

### 2.1. The Construction of pUbi: OsNPF7.6 Transgenic Rice

The ORF of *OsNPF7.6* (*LOC_Os04g50930*) was amplified from the full-length cDNA of rice *japonica* cv. ‘Nipponbare’, primer pairs were shown in Supplementary Table S1. PrimeSTAR HS DNA Polymerase (TaKaRa Biotechnology Co., Ltd., Dalian, China) was employed during the polymerase chain reaction (PCR). The parameters for PCR were 95 °C for 5 min, 94 °C for 30 s, 56 °C for 1 min (30 cycles), and 72 °C for 10 min. The PCR products were then ligated into the pMD19-T vector independently. The correct sequence fragment was ligated to the expression vector pTCK303 to obtain the *pUbi:OsNPF7.6* fusion vector. According to the previous description, the vector was introduced into the *Agrobacterium tumefaciens* strain, EHA105 via electroporation and subsequently transformed into Zhonghua 11 rice (WT) [3].

### 2.2. Identification of Positive Seedlings of Transgenic Lines

Through the application of the CATB method, the genomic DNA of T_2_ generation transgenic and wild-type plants was extracted [3] for PCR amplification of hygromycin fragments to identify positive seedlings, with primer pairs shown in Supplementary Table S2. The parameters for PCR were 95 °C for 3 min, 95 °C for 30 s, 55 °C for 30 s (30 cycles), and 72 °C for 10 min. Gel electrophoresis was used to show PCR products.

### 2.3. qRT-PCR

Following the previously reported method, plant genomic RNA extraction and gene expression analysis was conducted [3]. The primers for qRT-PCR were demonstrated in Table S3. 

### 2.4. Subcellular Localization of OsNPF7.6

The coding sequence of *OsNPF7.6* for subcellular localization was amplified using the pMD19-T vector and ligated into pSAT6A-GFP [33] with the correct direction. The *OsNPF7.6-GFP* vector was transformed into Arabidopsis mesophyll protoplasts and identified by a laser scanning microscope (LSM410; Carl Zeiss, Jena, Germany). Plasma membrane dye FMTM4-64FX (Invitrogen, Life Technologies, Carlsbad, CA, USA) was used as marker.

### 2.5. Plant Growth Conditions 

The *pUbi:OsNPF7.6* transgenic plants and WT were planted in three plots, which had 180 kg N/ha. The plot size was 2 m × 2.5 m, and seedlings were planted in a 10 × 10 array. During the stage of flowering and maturation, the samples of T_3_ generation were collected for further analysis.

The total nitrogen was analyzed as stated by Chen et al. [3]. According to our previously reported method, total nitrogen accumulation at the anthesis stage, total nitrogen accumulation at maturity stage, grain nitrogen accumulation at maturity, nitrogen translocation, nitrogen translocation efficiency, post-anthesis nitrogen uptake, nitrogen harvest index, as well as agronomic NUE were calculated [3]. Briefly, the calculation formulae are as follows. Nitrogen translocation (g/m^2^) = total nitrogen accumulation at anthesis − (total nitrogen accumulation at maturity − grain nitrogen accumulation at maturity); nitrogen translocation efficiency (%) = (nitrogen translocation/total nitrogen accumulation at anthesis) × 100%; post-anthesis nitrogen uptake (g/m^2^) = total nitrogen accumulation at maturity − total nitrogen accumulation at anthesis; the nitrogen harvest index (%) = grain nitrogen accumulation at maturity/total nitrogen accumulation at maturity. In addition, agronomic NUE (g/g) = grain yield/N supply.

### 2.6. Determination of ^15^N Influx Rates in Roots

Rice seedlings were planted in 1 mM NH_4_^+^ within a period of 3 weeks and subsequently subject to nitrogen starvation for 3 days for the root ^15^N uptake experiment. Initially, the plants were rinsed with 0.1 mM CaSO_4_ for 1 min, which were later transferred to either 0.5 mM ^15^NO_3_^−^ or 2 mM ^15^NO_3_^−^ (atom % ^15^N: 99%) solution for 5 min. Then rinsed the plants once more with 0.1 mM CaSO_4_ for 1 min. Based on our previously reported method, ^15^N influx rates were calculated [3]. 

### 2.7. Statistical Analysis

In order to perform statistical analysis, we used the single factor analysis of variance (ANOVA) and Tukey’s test in this study. The IBM SPSS Statistics 20 software (SPSS Inc., Chicago, IL, USA) was adopted for all statistical analysis. 

## 3. Results

### 3.1. OsNPF7.6 Was Induced to Be Expressed by Nitrate and Localizes in the Plasma Membrane

**Figure 1:**
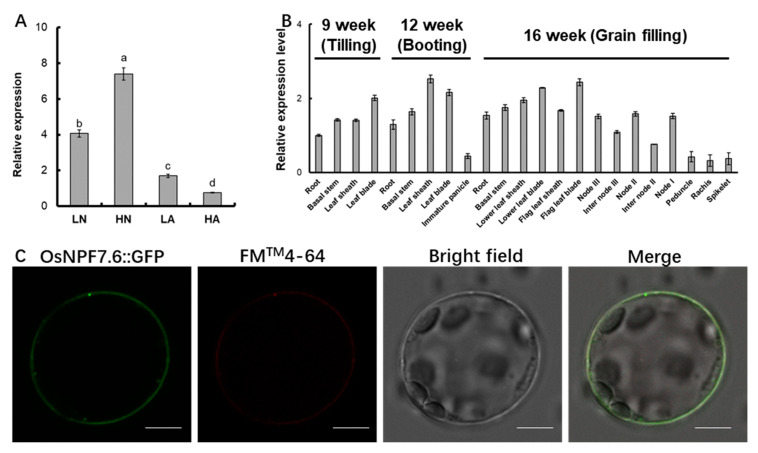
Expression pattern and subcellular localization of OsNPF7.6. (**A**) *OsNPF7.6* expression levels in roots of rice (cv. Nipponbare) under varying nitrogen supplies. The seedlings were grown in IRRI nutrient solution including 1 mM NH_4_^+^ for a period of 2 weeks, later moved to N-free solution for 3 d and then resupplied with NH_4_^+^ or NO_3_^−^ solution for 24 h. LN, 0.5 mmol/L NO_3_^−^ HN, 2.5 mmol/L NO_3_^−^; LA, 0.5 mmol/L NH_4_^+^; HA, 2.5 mmol/L NH_4_^+^. Values indicated ± SE (*n* = 3). The varying letters (a, b, c, d) suggest an obvious change under varying nitrogen supplies. (*p* < 0.05, one-way ANOVA) (**B**) Expression levels of *OsNPF7.6* in different organs at varying growth stages. Samples were taken from rice (cv. Nipponbare) planted in a paddy field. Values mean ± SE (*n* = 3). (**C**) *OsNPF7.6:GFP* vector was transformed into Arabidopsis mesophyll protoplasts and detected by laser scanning microscope (LSM410; Carl Zeiss, Germany). Scale bars = 10 μm.

The *OsNPF7.6* was localized on chromosome 4 with six exons and five introns (Figure S1). Under different nitrogen source treatments, the *OsNPF7.6* had the highest expression under 2.5 mM NO_3_^−^ conditions, followed by 0.5 mM NO_3_^−^ conditions, and the weakest expression under 2.5 mM NH_4_^+^ conditions (Figure 1A). The expression pattern of different sites at different fertility stages revealed that *OsNPF7.6* was constitutively expressed in rice at all sites at all times (Figure 1B). OsNPF7.6 had 10 transmembrane structures (Figure S2). In order to detect the subcellular localization of the OsNPF7.6 protein, an expression vector containing the *OsNPF7.6-GFP* fusion gene initiated by cauliflower mosaic virus 35S promoter was constructed and transformed into *Arabidopsis thaliana* protoplasts. After overnight transformation, the expression of OsNPF7.6-GFP in protoplasts was observed by confocal microscopy. *OsNPF7.6-GFP* fluorescence was completely merged with plasma membrane dye FM^TM^4-64FX (Figure 1C), and OsNPF7.6 was identified as a plasma membrane localization protein.

### 3.2. Acquisition of OsNPF7.6 Overexpression Lines

The *pUbi: OsNPF7.6* fusion vector was introduced into *Agrobacterium tumefaciens* strain EHA105 via electroporation and subsequently transformed into Zhonghua 11. A total of 72 transgenic seedlings from 10 strains were available, and 32 transgenic seedlings from eight strains survived after 7 days of screening with 200 mg/L hygromycin solution. The DNA of these 32 strains was extracted, the hygromycin fragments were amplified by PCR, and the target bands were detectable in all 32 transgenic seedlings. The positive rate of transgenic seedlings was 44.4%.

**Figure 2:**
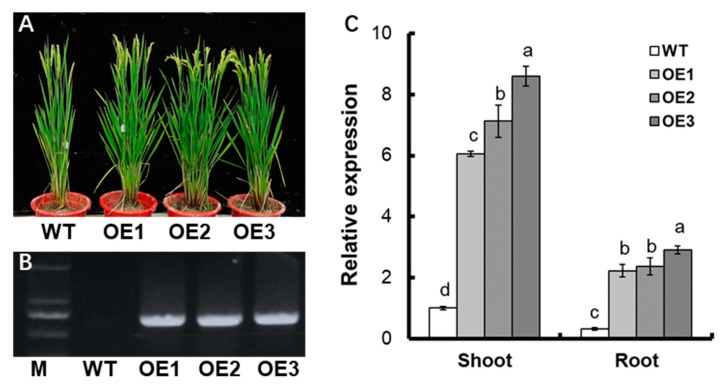
Identification of transgenic lines. (**A**) Phenotype of WT and *pUbi:OsNPF7.6* transgenic plants (OE1, OE2 and OE3). (**B**) The hygromycin fragment was amplified from the genomic DNA of T2 generation transgenic plants and WT. M represents a 2000 base pair (bp) DNA ladder. (**C**) qRT-PCR analysis the expression of *OsNPF7.6*. RNA was extracted from shoot and root. Values indicated ± SE (*n* = 3). The varying letters (a, b, c, d) suggest an obvious change between the transgenic line and the WT. (*p* < 0.05, one-way ANOVA).

Three transgenic strains were selected for T2 generation (Figure 2A), and then positive seedlings were verified by PCR amplification of hygromycin fragments (Figure 2B). qRT-PCR revealed that the expression level of *OsNPF7.6* increased about 7-fold in the shoot and root of transgenic lines relative to WT (Figure 2C).

### 3.3. Expression of OsNPF7.6 Increased ^15^NO_3_^−^ Influx Rates in Rice

**Figure 3:**
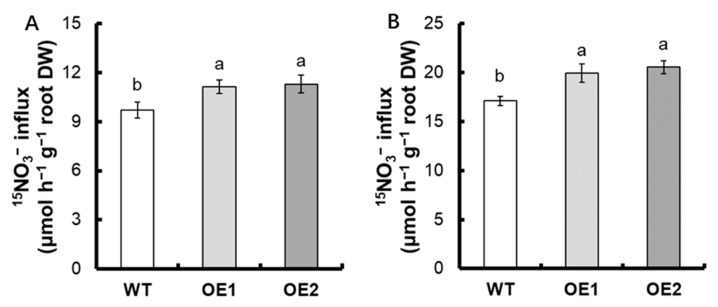
^15^NO_3_^−^ influx rates were measured with the use of ^15^N−enriched sources. WT and transgenic seedlings were grown in 1 mM NH_4_^+^ for 3 weeks and then were treated with nitrogen starvation for 3 days. ^15^N influx rates were measured at (**A**) 0.5 mM ^15^NO_3_^−^ or (**B**) 2.5 mM NO_3_^−^ for 5 min. DW, dry weight. Error bars: SE (*n* = 4). The varying letters (a, b) suggest an obvious change between the transgenic line and the WT. (*p* < 0.05, one-way ANOVA).

To evaluate the effect of *pUbi: OsNPF7.6* expression on root NO_3_^−^ influx into intact plants, seedlings of transgenic lines and WT were exposed to 0.5 mM ^15^NO_3_^−^ or 2.5 mM ^15^NO_3_^−^ for 5 min for short-term NO_3_^−^ and NH_4_^+^ absorption. The influx rates of ^15^NO_3_^−^ of *pUbi: OsNPF7.6* transgenic lines increased by 15.4% and 18.3%, respectively, compared with WT under 0.5 mM ^15^NO_3_^−^ and 2.5 mM ^15^NO_3_^−^ supply (Figure 3A,B).

### 3.4. Effects on Agronomic Traits of Rice after Overexpression of OsNPF7.6

**Figure 4:**
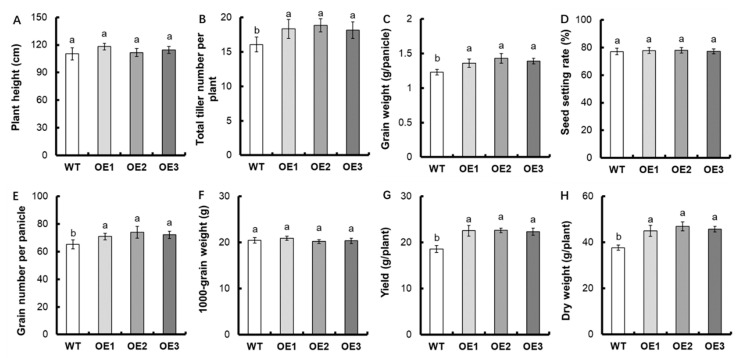
Comparison of agronomic traits between transgenic lines and WT. (**A**) Plant height, (**B**) Total tiller number per plant, (**C**) Grain weight per panicle, (**D**) Seed setting rate, (**E**) Grain number per panicle, (**F**) 1000-grain weight, (**G**) Grain yield, (**H**) Dry weight. Statistical analysis of data from T_3_ generation, *n* = 3. The varying letters (a, b) suggest an obvious change between the transgenic line and the WT. (*p* < 0.05, one-way ANOVA).

In order to analyze the effects of *OsNPF7.6* overexpression on rice growth and yield, the current work attempted to investigate the agronomic traits of the transgenic strains. The plant height, seed setting rate, and 1000-grain weight of the *OsNPF7.6* overexpression strains were not substantially different from WT (*p* > 0.05, Figure 4A,D,F). Compared to WT, the total tiller number per plant, grain weight per panicle, as well as grain number per panicle of *OsNPF7.6* overexpression strains increased by 14.8%, 13.3%, and 10.9%, respectively (Figure 4B,C,E). Ultimately, the *OsNPF7.6* overexpression strains improved grain production and dry weight by 21.0% and 21.7%, respectively (Figure 4G,H).

### 3.5. Effects on Nitrogen Utilization in Rice after Expression of OsNPF7.6

**Figure 5:**
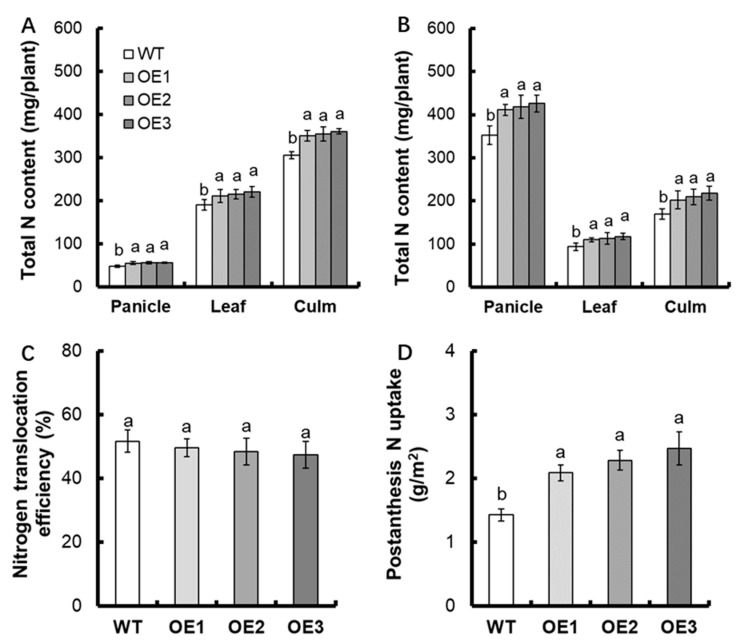
Total nitrogen content in different parts of transgenic lines at the stage of anthesis and maturity. (**A**) Anthesis stage, (**B**) Maturity stage. Comparison of (**C**) nitrogen translocation efficiency and (**D**) post-anthesis nitrogen uptake between WT and transgenic lines. Statistical analysis of data from T3 generation, *n* = 3. The varying letters (a, b) suggest an obvious change between the transgenic line and the WT. (*p* < 0.05, one-way ANOVA).

The dry matter production in plants per unit of applied nitrogen is also known as “nutrient utilization efficiency” and refers to the transfer of nitrogen to plants organs and yield [5]. In this study, we tested the total nitrogen content of the T_3_ generation transgenic lines during the anthesis and maturity stages. At the anthesis stage, total nitrogen accumulated mostly in the leaf and culm. Compared with WT, we found that at the anthesis stage, the total nitrogen content of panicles, leaves, and culms in transgenic lines increased by 16.9%, 13.2%, and 16.3%, respectively (Figure 5A). With the reproductive growth, the proportion of nitrogen content in spike to total nitrogen content of plant begins to increase. The results showed that the total nitrogen content of panicles, leaves, and culms in transgenic lines elevated by 18.7%%, 21.1%, and 24.1%, separately at maturity stage (Figure 5B).

**Figure 6:**
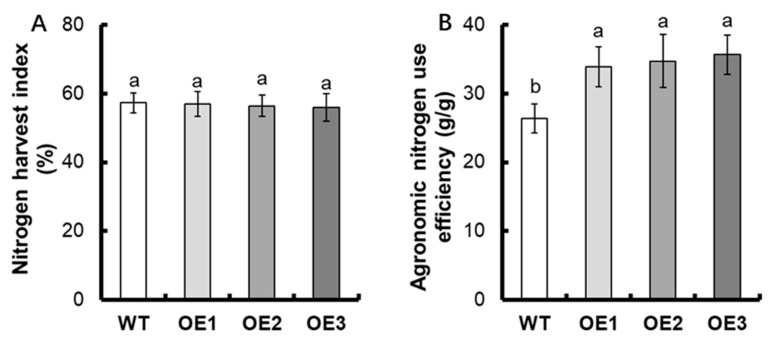
Comparison of NUE between WT and transgenic lines. (**A**) Nitrogen harvest index, (**B**) Agronomic nitrogen use efficiency. Statistical analysis of data from T3 generation, *n* = 3. The varying letters (a, b) suggest an obvious change between the transgenic line and the WT. (*p* < 0.05, one-way ANOVA).

For the NUE, no obvious difference in nitrogen translocation efficiency between transgenic lines and WT was found (Figure 5C). Compared with WT, the post-anthesis nitrogen uptake of transgenic lines increased by about 59.9% (Figure 5D). There was no evident divergence in the nitrogen harvest index between transgenic lines and WT (Figure 6A). The agronomic NUE of transgenic lines elevated by about 20.9% (Figure 6B).

## 4. Discussion

Rice contains 93 NPF family members [22]. As the necessary macronutrient for plant growth and development, nitrogen can limit crop productivity when it is scarce in the field [5]. Increasing the uptake and accumulation of nitrogen is the main way to improve the NUE and yield of rice.

Previous research has shown that *OsNPF2.4* [23], *OsNPF5.16* [24], *OsNPF6.1* [24], *OsNPF6.3* [25], *OsNPF6.5* (*OsNRT1.1b*) [26], *OsNPF7.1* [27], *OsNPF7.2* [28], *OsNPF7.4* [24,27], and *OsNPF7.7* [34] are involved in the NO_3_^−^ uptake process. *OsNPF4.5* exerts an important function in the acquisition of nitrate by the arbuscular mycorrhizal symbiosis pathway [25]. The *OsNRT1.1b* (*OsNPF6.5*) *indica* variant improves nitrate uptake and could elevate the NUE [26]. *OsNPF7.2* is expressed mostly in thick-walled cells of the root elongation and maturation zones, the cortex, and the mid-column. It is involved in NO_3_^−^ uptake as well as NO_3_^−^ partitioning in different sections of the root [28]. *OsNPF7.7* contains two variable splices: *OsNPF7.7-1* (encoding the longer product) and *OsNPF7.7-2* (encoding the shorter product). *OsNPF7.7* is located in the cell membrane (*OsNPF7.7-1*) and vesicle membrane (*OsNPF7.7-2*), with higher expression levels boosting the uptake of NO_3_^−^ and NH_4_^+^, respectively (Hu et al., 2018). Nitrate promoted the expression of *OsNPF7.6* in rice roots (Figure 1A), and subcellular localization demonstrated that OsNPF7.6 was located in the plasma membrane (Figure 1C). Under the supply of 0.5 mM ^15^NO_3_^−^ and 2.5 mM ^15^NO_3_^−^, the influx rates of ^15^NO3^−^ of *pUbi:OsNPF7.6* transgenic lines increased by 15.4% and 18.3%, respectively, compared with WT (Figure 3A,B). These results illustrate that *OsNPF7.6*, a member of the *NFP7* subfamily, is also involved in nitrate uptake by the rice root system.

Nitrogen is crucial for crop growth and development, notably for the creation of tillers, which belongs to one of the most crucial elements determining grain yield in rice [25]. *OsNPF7.1*, *OsNPF7.2*, *OsNPF7.3*, *OsNPF7.4*, and *OsNPF7.7* are all members of the NFP7 subfamily that contribute to the development of rice tiller buds, which in turn influences rice tillering. *OsNPF7.1* and *OsNPF7.4* are two of them that, in response to different nitrogen supply levels, exhibit opposing expression patterns in tiller buds and have enhanced and inhibited effects on tiller bud growth and development, respectively. [27]. OsNPF7.2 could control tiller bud growth and root development through the regulation of cytokinin and cell cycle in plants [25,35,36]. OsNPF7.3 overexpression promotes growth, tiller number, grain yield, and grain nitrogen content [37,38]. We also found that overexpression of *OsNPF7.6* increased the total tiller number per plant in rice (Figure 4B). Compared with WT, the grain weight and grain number of per panicle increased by 13.3% and 10.9%, respectively, in the *OsNPF7.6* overexpression strains (Figure 4C,E). Consequently, the grain yield and dry weight of the *OsNPF7.6* overexpression strains increased by 21.0% and 21.7%, respectively (Figure 4G,H).

A series of studies have shown that nitrate is necessary for nitrogen nutrition in rice. It is found that nitrate allocation and the divergent nitrate use efficiency between *indica* and *japonica* rice can be mediated by the nitrate transporter OsNPF7.9 [39]. *OsNPF6.1^HapB^* is the rare natural allele, which can control nitrate uptake and NUE [32]. OsNRT1.1A/OsNPF6.3 exerts the vital function in nitrogen uptake, transport, and assimilation [25]. In addition, the overexpression of *OsNAR2.1* is shown to improve rice yield and NUE [2]. Following the introgression of both the *indica OsNRT1.1B* and *OsNR2* alleles into *japonica* accessions, the grain yield and NUE could be deeply enhanced [31]. We also found that overexpression of *OsNPF7.6* can increase the agronomic NUE in rice (Figure 6B). Although the nitrogen accumulation of *OsNPF7.6* overexpression lines increased significantly (Figure 5A,B), there existed no obvious difference in nitrogen translocation efficiency and nitrogen harvest index between transgenic lines and WT (Figure 5C and Figure 6A). Thus, the overexpression of *OsNPF7.6* does not affect nitrogen distribution in rice.

In conclusion, OsNPF7.6 is a nitrate transporter located in the plasma membrane. Overexpression of *OsNPF7.6* can increase the nitrate uptake rate of rice. Field experiments showed that overexpression of *OsNPF7.6* elevated the total tiller number per plant and the grain weight per panicle, which could thus enhance grain yield and agronomic NUE in rice.

## Data Availability

All of the data generated or analyzed during this study are included in this published article.

## Supplementary Materials

The following are available online at https://www.mdpi.com/article/10.3390/life12121981/s1, Table S1. Primers were used to amplify the OsNPF7.6 open reading frame, Table S2. Primers were used to amplify the hygromycin fragment, Table S3. Primers were used to detect the expression of OsActin, OsNPF7.6, Figure S1. Analysis of OsNPF7.6 gene structure. Checked through the Rice Genome Annotation Project (http://rice.uga.edu/index.shtml (accessed on 10 August 2022)), Figure S2. Analysis of transmembrane domains of OsNPF7.6. Predicted by TMHMM 2.0 (http://www.cbs.dtu.dk/services/TMHMM/ (accessed on 10 August 2022)).
