# Supplementary material for: Overexpression of Nitrate Transporter 1/Peptide Gene OsNPF7.6 Increases Rice Yield and Nitrogen Use Efficiency
Source: Life (Basel). 2022 Nov 26;12(12):1981. doi: 10.3390/life12121981 (PMC9786031; doi:10.3390/life12121981)
Supplement: Supplementary file 1 [file life-12-01981-s001.zip › life-1993786-supplementary.pptx]

## Slide 1
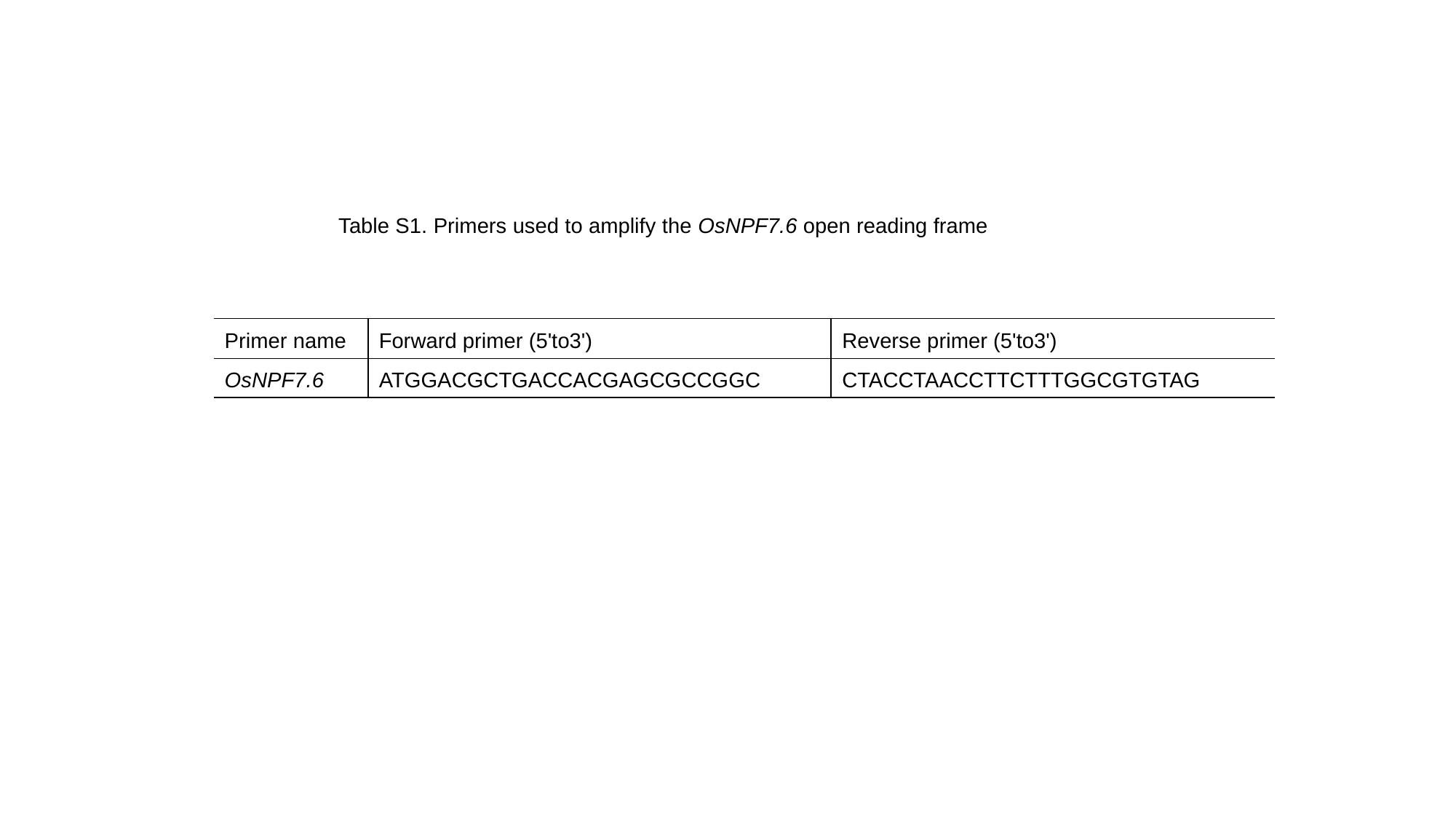

Table S1. Primers used to amplify the OsNPF7.6 open reading frame
| Primer name | Forward primer (5'to3') | Reverse primer (5'to3') |
| --- | --- | --- |
| OsNPF7.6 | ATGGACGCTGACCACGAGCGCCGGC | CTACCTAACCTTCTTTGGCGTGTAG |

## Slide 2
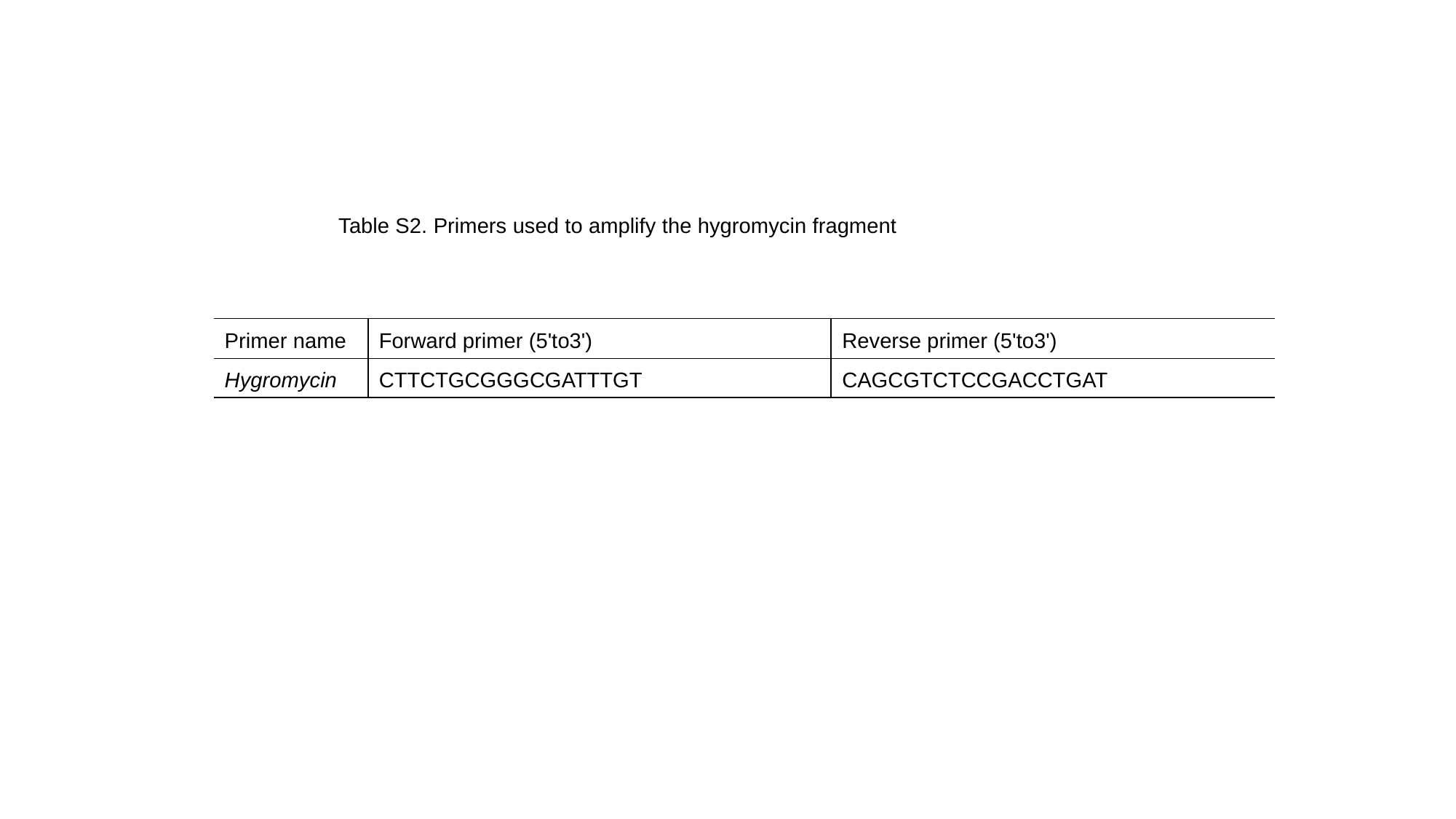

Table S2. Primers used to amplify the hygromycin fragment
| Primer name | Forward primer (5'to3') | Reverse primer (5'to3') |
| --- | --- | --- |
| Hygromycin | CTTCTGCGGGCGATTTGT | CAGCGTCTCCGACCTGAT |

## Slide 3
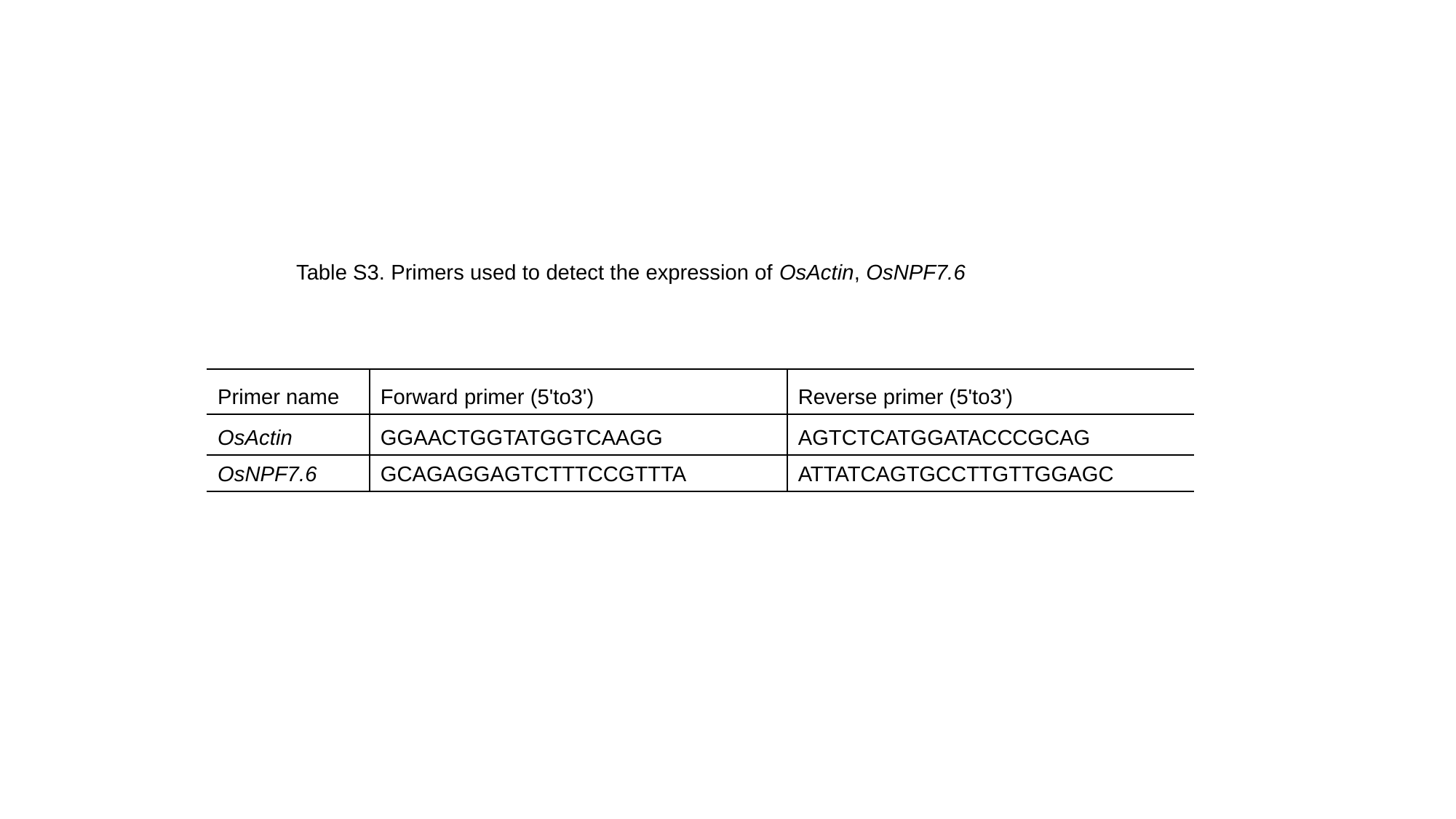

Table S3. Primers used to detect the expression of OsActin, OsNPF7.6
| Primer name | Forward primer (5'to3') | Reverse primer (5'to3') |
| --- | --- | --- |
| OsActin | GGAACTGGTATGGTCAAGG | AGTCTCATGGATACCCGCAG |
| OsNPF7.6 | GCAGAGGAGTCTTTCCGTTTA | ATTATCAGTGCCTTGTTGGAGC |

## Slide 4
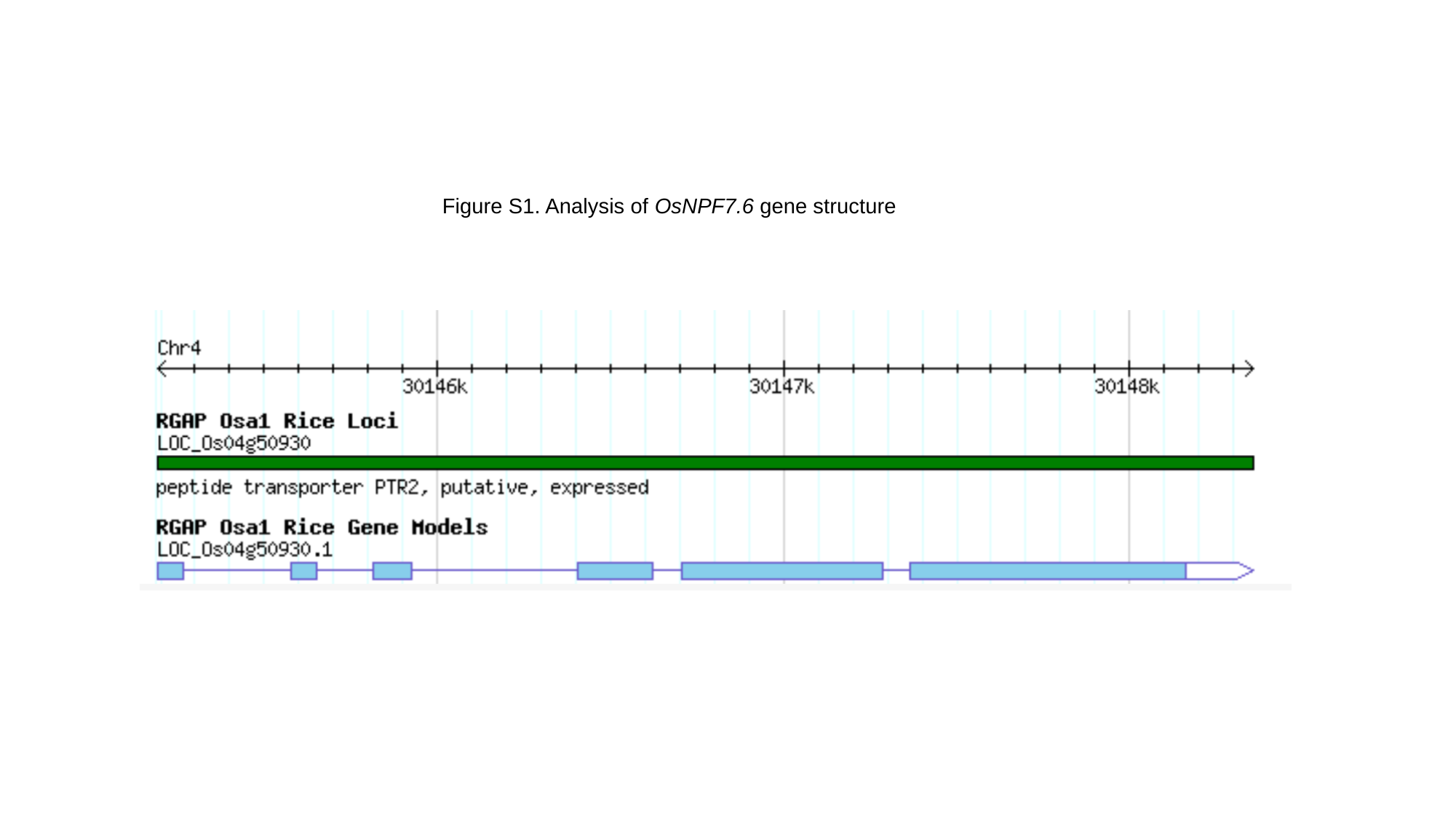

Figure S1. Analysis of OsNPF7.6 gene structure

## Slide 5
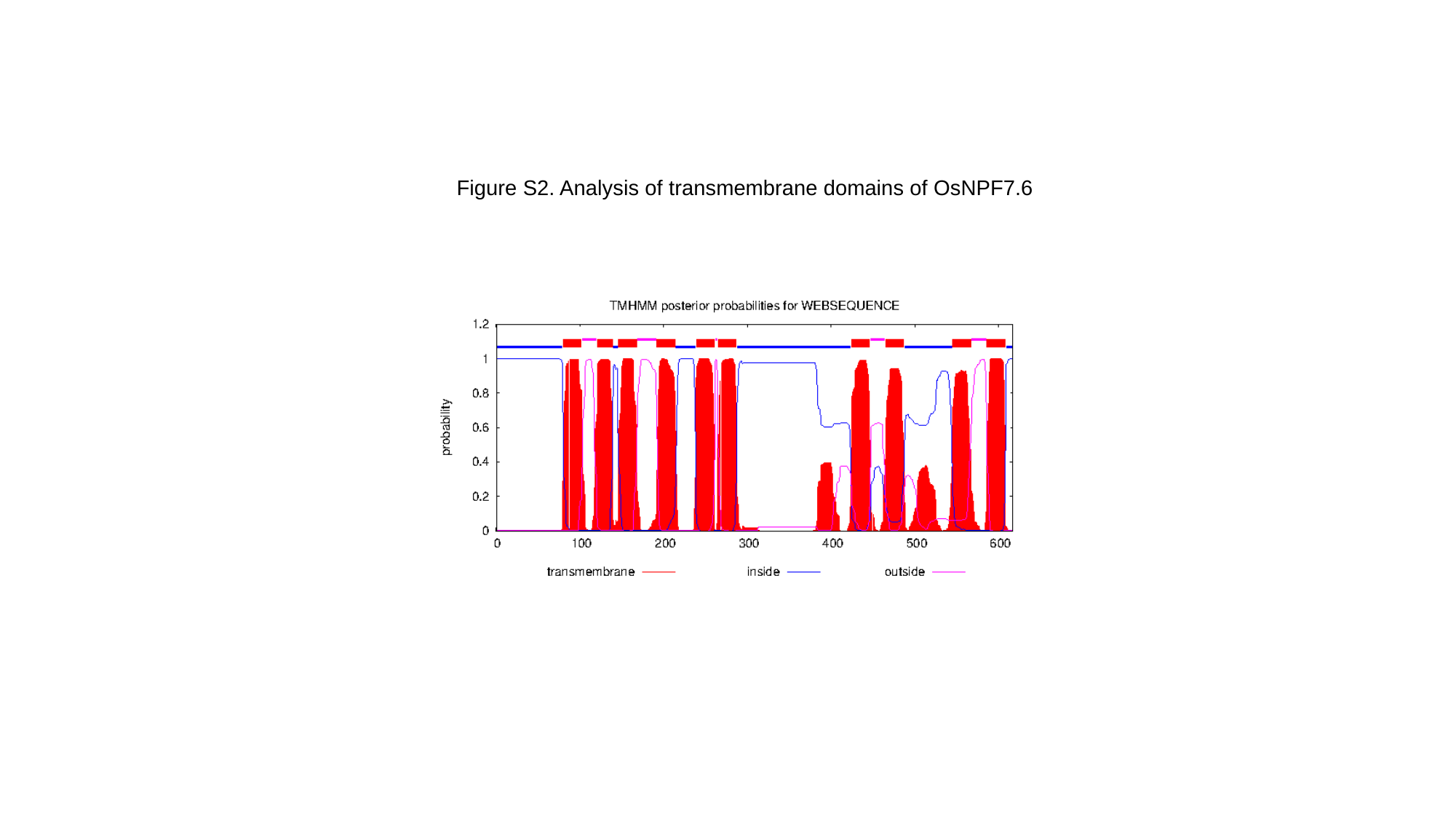

Figure S2. Analysis of transmembrane domains of OsNPF7.6
